# High‐Performance Flexible Sulfur Cathodes with Robust Electrode Skeletons Built by a Hierarchical Self‐Assembling Slurry

**DOI:** 10.1002/advs.202201881

**Published:** 2022-07-19

**Authors:** Zhengmin Zhang, Jiangyang Mo, Peng Yu, Lanxiang Feng, Yu Wang, Yuyuan Lu, Wei Yang

**Affiliations:** ^1^ College of Polymer Science and Engineering State Key Laboratory of Polymer Materials Engineering Sichuan University Chengdu Sichuan 610065 China; ^2^ State Key Laboratory of Polymer Physics and Chemistry Changchun Institute of Applied Chemistry Chinese Academy of Sciences Changchun Jilin 130022 China; ^3^ State key Laboratory of Biotherapy and Cancer Center West China Hospital Sichuan University Chengdu Sichuan 610041 China

**Keywords:** electrode skeleton structures, flexible lithium–sulfur batteries, hierarchical pores, phase separation, self‐assembling electrode slurry

## Abstract

The electrochemical performance of lithium–sulfur batteries is fundamentally determined by the structural and mechanical stability of their composite sulfur cathodes. However, the development of cost‐effective strategies for realizing robust hierarchical composite electrode structures remains highly challenging due to uncontrollable interactions among the components. The present work addresses this issue by proposing a type of self‐assembling electrode slurry based on a well‐designed two‐component (polyacrylonitrile and polyvinylpyrrolidone) polar binder system with carbon nanotubes that forms hierarchical porous structures via optimized water‐vapor‐induced phase separation. The electrode skeleton is a highly robust and flexible electron‐conductive network, and the porous structure provides hierarchical ion‐transport channels with strong polysulfide trapping capability. Composite sulfur cathodes prepared with a sulfur loading of 4.53 mg cm^−2^ realize a very stable specific capacity of 485 mAh g^−1^ at a current density of 3.74 mA cm^−2^ after 1000 cycles. Meanwhile, a composite sulfur cathode with a high sulfur loading of 14.5 mg cm^−2^ in a lithium–sulfur pouch cell provides good flexibility and delivers a high capacity of 600 mAh g^−1^ at a current density of 0.72 mA cm^−2^ for 78 cycles.

## Graphical abstract

A robust electrode skeleton with hierarchical pore system is built through the optimized vapor‐induced phase separation technology based on a well‐designed self‐assembling polymer blends slurry. Flexible sulfur cathodes with this electrode skeleton delivered a high specific capacity of 600 mAh g^−1^ for 78 cycles at 0.03 C with high sulfur loading of 14.5 mg cm^−2^.

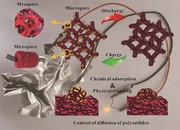

## 1 Introduction

Lithium–sulfur (Li–S) batteries are promising next‐generation electrical energy storage devices due to their high energy and power densities, low toxicity, low cost, and the high availability of sulfur.^[^ ^1^, ^2^, ^3^ ^]^ However, the practical applications of Li–S batteries are greatly limited by their low conductivity, Li‐polysulfide (LiPS) shuttle effects, and notable volume change during charge/discharge cycling.^[^ ^4^, ^5^ ^]^ Considerable work has been conducted to address these issues via the design and synthesis of sulfur‐based active materials (AMs) with rational chemical and physical structures in composite sulfur electrodes.^[^ ^6^, ^7^, ^8^ ^]^ This is a reasonable focus because sulfur‐based AM particles are the dominant components affecting the performance of composite sulfur electrodes. However, the high‐level structures of composite electrodes formed from all the components, denoted as assembly structures, are another key factor controlling the ion and electron transport capabilities of these electrodes, as well as their structural stability during cycling.^[^ ^9^, ^10^ ^]^ Assembly structures are physically similar to the quaternary structures well known in protein science, which are significant for facilitating their biological functionality. Therefore, efforts to address the limitations of Li–S batteries require the development of strategies for realizing well‐designed and controlled assembly structures in composite sulfur electrodes.^[^ ^11^, ^12^ ^]^

Conventional approaches for controlling the assembly structures of composite electrodes involve the use of a polyvinylidene fluoride binder together with carbon black or multi‐walled carbon nanotube (MWCNT) components to improve the electronic conductivity of the assembly structures. However, the component binding strength obtained is generally insufficient for ensuring the stability of composite sulfur electrodes during prolonged charge/discharge cycling. This issue can be addressed to some extent by an appropriate calendering treatment, which increases particle contact by decreasing the electrode porosity. Moreover, efforts have been made toward the development of high‐performance conductive agents for increasing the electronic conductivity of assembly structures. For example, two‐dimensional (2D) graphene has been demonstrated to represent a superior conductive agent for developing high‐performance composite sulfur cathodes due to its high conductivity and *π*–*π* interactions supporting long‐range conductive connections.^[^ ^13^, ^14^, ^15^ ^]^ Moreover, these studies have demonstrated that graphene can help trap dissolved polysulfides physically, and thereby manage LiPS shuttle effects. However, a close stacking of graphene can hinder electrolyte infiltration, and thereby limit ion transport.^[^ ^16^ ^]^

The limitations associated with an exclusive focus on increasing the electronic conductivity of composite sulfur electrodes has led recent studies to pay more attention to build robust functional assembly structures with not only high electronic conductivity, but also with a rational porous configuration that supports fast ion conduction. These efforts include the use of sacrificing templates to control the pore structure inside composite electrodes and the use of conductive foams and carbon‐based fabrics as electrode skeletons.^[^ ^17^, ^18^, ^19^, ^20^ ^]^ Past studies have demonstrated that macroscale pores introduced into electrode skeletons are beneficial for ensuring electrolyte infiltration and fast ion transport.^[^ ^21^, ^22^, ^23^ ^]^ In addition, the introduction of microscale pores into electrode skeletons prevents the loss of AM particles and reduces the impacts of LiPS shuttle effects.^[^ ^24^, ^25^, ^26^ ^]^ Moreover, the high specific surface area (SSA) of porous skeletons and polar groups in the polymer chains of binders also provide strong physicochemical adsorption capabilities for trapping polysulfides.^[^ ^27^, ^28^ ^]^ Despite the success of these past efforts, a number of issues remain unresolved. For example, the means of rationally designing electrode skeletons that support the high electronic and ionic transport, good polysulfide capture, and high structural stability requirements of the composite sulfur cathodes remain poorly understood. Meanwhile, the development of new cost‐effective strategies is still urgently required for realizing desired electrode skeleton structures.

**Figure 1:**
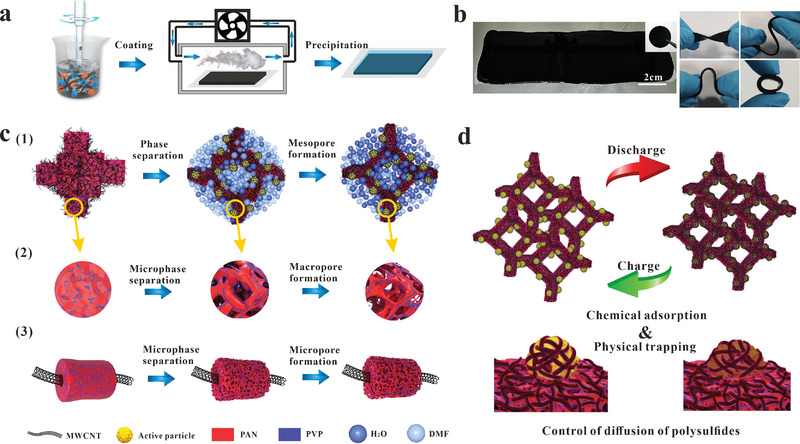
Self‐assembling slurry design for building the robust hierarchical porous electrode skeletons employed in composite sulfur cathodes and proposed hierarchical electrode skeleton self‐assembling mechanism: a) preparation of electrode skeletons by the self‐assembling slurry. b) Images of the slurry during water‐vapor‐induced self‐assembly (left) and the flexibility demonstration of final flexible composite sulfur cathode (right). c) Schematic illustration of the hierarchical self‐assembling process induced by gaseous water molecules. d) Designed functions of the electrode skeleton for addressing the critical issues of volume change and LiPS shuttle effects in composite sulfur electrodes.

To address the above issues, in simple polymer blends, this work optimized a hierarchical self‐assembling electrode slurry based on a specially designed two‐component binder system (polyacrylonitrile (PAN) and poly(vinyl pyrrolidone) (PVP)) as well as the phase separation process to achieve hierarchical porous structures for particularly flexible electrode applications. As illustrated in **Figure** **1**a, the self‐assembling behavior of the electrode slurry is realized by phase separation induced by water vapor. The electrode skeleton offers good structural stability, high electronic conductivity, and hierarchical porous structures that support fast ion transportation. As discussed above, the macropores in the skeleton are beneficial for electrolyte infiltration and fast ion transportation,^[^ ^21^, ^22^, ^23^ ^]^ while the micropores in the skeleton prevent the loss of AM and reduce the impacts of LiPS shuttle effects.^[^ ^24^, ^25^, ^26^ ^]^ Moreover, the porous skeleton also provides a high SSA and polar groups from the polymer chains that maintain a strong physicochemical adsorption capability for polysulfide trapping.^[^ ^27^, ^28^ ^]^ The sulfur‐based AM particles are integrated into the skeleton via a self‐wrapping process, as revealed in a previous study.^[^ ^29^ ^]^ Results demonstrate that Li–S cells with sulfur electrodes fabricated using the specially designed electrode slurry exhibit stable electrochemical performance. Specifically, the cells obtain a stable capacity of 485 mAh g^−1^ at a current density of 0.5 C even after 1000 cycles, corresponding to a capacity decay of only 0.036% per cycle. In addition to improving the cycling performance, the robust self‐assembled electrode skeleton also demonstrates good mechanical flexibility. The above function‐integrated electrode skeleton design via the self‐assembling slurry may provide a scalable and promising solution for regulating assembly structures on a composite level, which is critical for developing composite electrodes with high‐capacity AMs or good flexibility.

## 2 Results and Discussion

In Figure 1a, the two polar PAN and PVP polymers are dissolved in *N*,*N*‐dimethylformamide (DMF) to form a uniformly mixed binder solution. The porous structures of the electrode skeleton were found to be sensitive to the composition of the slurry as well as environmental conditions. The four critical factors affecting the pore self‐assembling process were relative humidity, temperature, polymer solution composition, and the flow rate of humidified air. Therefore, a special enclosure was developed to control the environment (Figure S1, Supporting Information). Details regarding the process applied for determining the optimal environmental conditions and PAN:PVP ratio are summarized in the Supporting Information (Section S1 and Figures S2–S8, Supporting Information). Briefly, the optimal set of factors, including *humidity* = 60%, *temperature* = 25 °C, and 5 wt% for the composite PAN/PVP polymer concentration. Meanwhile, PAN:PVP ratio of 7:1 was applied as the optimal ratio.

The solid components in the slurry were observed to aggregate quickly when exposed to water vapor for about 2 min, as shown in Figure 1b, which also generated a clear solvent layer along the boundary of the slurry. Moreover, the 3D electrode skeleton is mechanically robust, as demonstrated by the ability to fold and twist the wet electrode without breaking. Among samples with different ratios of PAN and PVP, PAN/PVP/MWCNT with PAN and PVP ratio of 7:1 (C/A/V‐7:1) skeleton possesses the highest tensile strength of ≈0.44±0.02 MPa and the largest strain‐at‐failure of ≈28.9±7.6% (Figure S7a,b, Supporting Information). C/A/V‐7:1 skeleton also shows a high storage modulus and possesses better robustness with temperature (Figure S7c,d, Supporting Information). The electrode skeleton maintains its structural integrity after drying and cutting into circular samples, as shown in the inset of Figure 1b, although it is as flexible as when observed in its wet state. During this step, the free PAN chain may first experience phase separation due to its poor affinity to water molecules, which can be expected to lead to the development of interior macropores. As illustrated in Figure 1c, an increasing concentration of water molecules absorbed by the slurry produces a solvent environment that is increasingly unamiable to PAN. Therefore, the PAN chains absorbed by PVP may experience microphase separation and generate mesopores. Meanwhile, during the above self‐assembling process, the PAN/PVP chains attached onto conductive nanomaterials (MWCNTs) also experience phase‐separation on the MWCNT surfaces, leading to even smaller porous structures (micropores) on the surface. The micropores should be of great interest for trapping the polysulfides.^[^ ^24^, ^25^, ^26^ ^]^ In addition to the high surface area of the hierarchical porous structures, the PVP and PAN polymer chains also have strong polysulfide absorption capability.^[^ ^27^, ^28^ ^]^ As a result, the porous electrode skeleton is electrochemically enhanced for LiPS trapping, as illustrated by Figure 1d.

**Figure 2:**
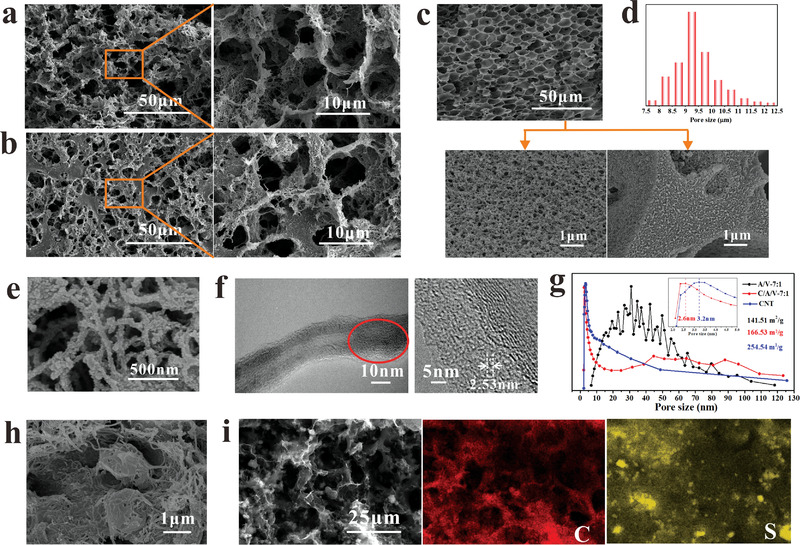
Hierarchical pore structures of the A/V‐7:1 (pure PAN/PVP), C/A/V‐7:1 (with MWCNTs), and SC/A/V‐7:1 electrode skeletons produced under optimal conditions: SEM images of a) a CA/V‐7:1 free surface and b) cross‐sectional surface. c) SEM images of an A/V‐7:1 surface, and a corresponding pore surface and cross‐section of a pore wall. d) Macroscale pore size distribution of A/V‐7:1 based on BET analysis. e) SEM and f) TEM images of MWCNT surfaces with A/V‐7:1 coatings. g) Pore size distributions of A/V‐7:1, C/A/V‐7:1, and pure MWCNTs. h) SEM image of the composite SC/A/V‐7:1 cathode with graphene@sulfur particles encased in the C/A/V‐7:1 network. i) EDS mapping of the carbon (C) and sulfur (S) concentrations over a section of the SC/A/V‐7:1 cathode.

The hierarchical pore structures of the C/A/V‐7:1 electrode skeleton obtained under the optimal conditions with MWCNTs can be characterized according to the scanning electron microscope (SEM) images given in **Figure** **2**a,b. The images present a uniform macroscale pore structure with an average size of about 10 µm throughout the skeleton. However, the addition of MWCNTs makes the mesoscale pores of the skeleton difficult to identify. Therefore, both the macroscale and mesoscale pore structures were evaluated based on the pure PAN/PVP with PAN and PVP ratio of 7:1 (A/V‐7:1) sample processed without MWCNTs. The low‐magnification SEM image in Figure 2c indicates that this sample presents a uniform macroscale pore structure, and the Brunner−Emmet−Teller (BET) analysis results in Figure 2d demonstrate that the skeleton has an average macroscale pore size of about 9 um, which is very close to that observed for the C/A/V‐7:1 sample. At the same time, the high‐magnification SEM image in Figure 2c exhibits mesoscale pores with pore sizes in the range of 15–100 nm, which can be potentially attributed to microscale phase separations between PAN and PVP. The SEM image in Figure 2e demonstrates that PAN/PVP binder adheres to the MWCNTs. Furthermore, the transmission electron microscope (TEM) image in Figure 2f demonstrates that the introduction of MWCNTs generates microscale pores on the MWCNT surfaces with pore sizes of about 2.5 nm, which is further demonstrated by the pore size distribution shown in Figure 2g for the C/A/V‐7:1 sample that provides an average microscale pore size of about 2.6 nm. Meanwhile, the mesoscale pore size of this sample ranges from 25 to 110 nm, which is similar to that observed for A/V‐7:1 prior to MWCNT addition. In addition, micropores are uniformly formed on the surface of CNT in C/A/V‐7:1 (Figure 2e,f). However, micropores were not found on CNT in sample C/A/V‐1:0 (Figure S9a, Supporting Information), and the micropores were closed and incomplete for the samples with other ratios of PAN:PVP (Figure S9b–d, Supporting Information). Moreover, the introduction of MWCNTs notably increases the specific surface area from 141.51 m^2^ g^−1^ for the pure A/V‐7:1 skeleton to 166.53 m^2^ g^−1^ for C/A/V‐7:1 by generating a higher concentration of mesopores and micropores. The SEM image of the SC/A/V‐7:1 electrode skeleton in Figure 2h presents well‐dispersed graphene@sulfur particles (structure of graphene@sulfur particles is shown in Figure S10, Supporting Information) encased in the skeleton. The energy dispersive X‐ray spectroscopy (EDS) mapping results in Figure 2i confirm that the sulfur‐based AM particles are attached and encased by the carbonaceous components of the skeleton.

**Figure 3:**
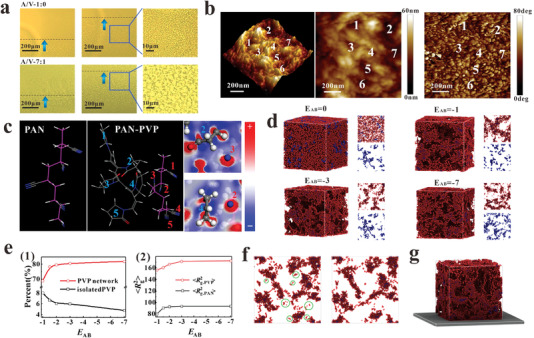
Experimental investigations of the phase separation process and simulation studies of PAN–PVP chain interactions and pore formation for binder systems: a) optical microscopy images of a single drop of the pure PAN (A/V‐1:0) binder solution (top) and the mixed A/V‐7:1 binder solution (bottom) applied to the surface of a clean glass slide under optimal environmental conditions after water vapor infiltration. b) AFM topography and phase mapping for the A/V‐7:1 electrode skeleton. c) The five structural units of PAN and PAN–PVP chain segments employed in MD simulations, and the charge distributions in the mixed system. d) Results of Monte Carlo simulations obtained for a system composed of polymer A (PAN) and polymer B (PVP) with different interaction energies *E*_AB_. e) Effects of *E*_AB_ on the ratio of network B to isolated B (left) and on the mean squared radius of gyration (〈*R*_g_^2^〉) of A and B (right). f) Cross‐sectional slices of polymer AB systems before (left) and after (right) skeleton formation (*E*_AB_ = −7). g) Simulated AB polymer system (*E*_AB_ = −7) in contact with an MWCNT layer given in gray, where the interaction energy between the polymer chains and the MWCNT layer varied from −2 to 0 with increasing distance of separation.

The formation mechanism of the above‐discussed hierarchical porous structure was investigated according to specially designed experiments and simulations based on the assumption that pores with multiple spatial scales form via microphase separations between the PAN, PVP, and DMF components in the slurry induced by the infiltration of water molecules, which has different impacts on the PAN and PVP components. Accordingly, the phase‐separation process was observed experimentally by applying a single drop each of the pure PAN (A/V‐1:0) and mixed A/V‐7:1 polymer binder solution to the surface of a clean glass slide under the optimal environmental conditions with a relative humidity of 60%, a temperature of 25 °C, and circulating flow, and examining the outcome of water vapor infiltration in situ by optical microscopy. Here, the edge of the droplet is most vulnerable to the infiltration of water vapor, and the binder solution evaporates faster at the edge than at the center of the droplet. Therefore, the skeleton will first form along the edge of the droplet, which makes this the frontline of skeleton formation (Figure S11, Supporting Information) and a good position for observing both the solution state and the phase‐separation state. As shown at the top of **Figure** **3**a, the A/V‐1:0 polymer binder was separated from the DMF solvent with the absorption of water vapor. Then, it gradually self‐assembled into a skeleton with relatively small pore sizes via a gelation process. In contrast, the image at the bottom of Figure 3a demonstrates that the addition of PVP increases the pore sizes of the formed skeleton. The phase separation process can be expected to differ substantially with the addition of PVP because PVP is dissolvable in both DMF and H_2_O. As a result, the PVP chains will maintain strong interactions with PAN chains during the phase separation process, and fewer PAN chains will be involved in skeleton formation, which can be expected to lead to increased pore size. In fact, this increasing pore size with increasing PVP content has been observed explicitly (Figure S4, Supporting Information).

The pore formation mechanism associated with the mixed A/V‐7:1 binder can be further evaluated according to the atomic force microscopy (AFM) topography and relative phase mapping results presented in Figure 3b. The figure presents seven mesopores identified within a 1 µm × 1 µm mapping area according to both the topography and phase images. Interestingly, the phase‐mapping image indicates the presence of two continuous phases around each mesopore position. Accordingly, the mesopores may have been mainly created by microscale phase separations between PAN and PVP. This possibility is supported by the fact that microscale phase separations are always observed in block copolymer systems, and incompatible chain blocks tend to form different microphases via self‐assembling processes.^[^ ^30^, ^31^ ^]^ However, preferential phase separation at the microscale may also be supported in the PAN/PVP system due to strong attractive interactions between PAN and PVP chains that may limit the extent to which these chains can be separated from each other.

The strength of interactions between PAN and PVP chains was evaluated according to the results of molecular dynamics (MD) simulations conducted with PAN chains, PVP chains, and mixed PAN and PVP chains composed of five structural units each.^[^ ^32^ ^]^

The PAN and mixed PAN/PVP chain systems are illustrated in Figure 3c. We note that the inclusion of the PVP chain changes the conformational structure of the PAN chain considerably, which is indicative of notable interactions between the two polymer chains. Moreover, the side groups of PAN and PVP chains tend to attract each other during the kinetic optimization process, which also indicates the strong interactions between PAN and PVP (Figure S12, Supporting Information). This was evaluated quantitatively according to the interaction energy (Δ*E*_PAN/PVP_) calculated using the following equation

(1) $$\Delta E_{\mathrm{PAN}/\mathrm{PVP}}\;=\;E_{\mathrm{PAN}/\mathrm{PVP}}\;-\;E_{\mathrm{PAN}}\;-\;E_{\mathrm{PVP}}\;$$

The results returned a large negative value of Δ*E*_PAN/PVP_ = −7.716 eV. The attractive interaction increases as the interaction energy becomes increasingly negative. Therefore, this value of Δ*E*_PAN/PVP_ is representative of a strong attractive interaction between PAN and PVP. The attractive interactions between the two polymers can be inferred from the charge distribution shown in Figure 3c, which is indicative of strong polar–polar attraction.

For a better understanding of the effects of PAN–PVP interaction on the formation of co‐continuous microphase structures, a lattice‐based Monte Carlo method^[^ ^33^, ^34^ ^]^ combined with the bond fluctuation model was employed to perform simulation studies. This combined model has been extensively applied to bulk polymer melts^[^ ^33^, ^34^, ^35^ ^]^ and polymer blends^[^ ^36^, ^37^, ^38^, ^39^ ^]^ due to its computational efficiency. The results are presented in Figure 3d, which was obtained for a system composed of polymer A (PAN), polymer B (PVP), and molecule C (DMF) with different interaction energies *E*_AB_ between polymers A and B in the range of 0 to −7*k*_B_ *T*, here *k*_B_ *T* is the unit of energy. The other interaction energies were set according to the energies obtained from MD simulations as *E*_AC_ = −0.1 and *E*_BC_ = −0.2, where *E*_AC_ and *E*_BC_ denote the interaction energies between A and DMF, and B and DMF. The results indicate that a continuous A phase is formed, but the regions of B phase are isolated in the absence of interactions between polymers A and B (i.e., *E*_AB_ = 0). However, both A and B generate separate regions of continuous phases with increasingly negative *E*_AB_, and these regions become increasingly uniform, while the average size of the intervening micropore regions decreases.

The results in Figure 3d were subjected to detailed analysis to evaluate the means by which A–B chain interactions affect microphase separation in the AB system. We first evaluate how A–B chain interactions affect the continuity of polymer B by comparing the ratio of the B phase forming a continuous microphase network to the isolated polymer B presented in Figure 3e as a function of the absolute value of *E*_AB_ (|*E*_AB_|). The results indicate that more than 80% of polymer B chains will be involved in the B‐phase network when |*E*_AB_| is approximately greater than 2. Meanwhile, the results in Figure 3e also indicate that the mean squared radius of gyration (〈*R*_g_^2^〉) values of polymers A and B both increase with increasing |*E*_AB_|, and quickly approach constant values. The microphase separation of the AB system at *E*_AB_ = −7 is further demonstrated in Figure 3f, which presents the cross‐sectional slices of polymer AB systems obtained before (left) and after (right) skeleton formation. With an increasing amount of antisolvent, the two co‐continuous microphases can deform, and a greater number of isolated A chains aggregate together prior to skeleton formation, as indicated by the circled areas in the figure. The lattice‐based Monte Carlo simulation result obtained for the AB polymer system (*E*_AB_ = −7) in contact with a continuous MWCNT layer is presented in Figure 3g, where the interaction energy between the polymer chains and the contacting surface of the MWCNT layer was varied from −2 to 0 with increasing distance of separation. Interestingly, the sizes of the observed micropores decreased with decreasing distance between the polymer chains and the contacting MWCNT surface. This finding indicates that attractive interactions between the polymers and MWCNTs decrease the size of microphase separations.

**Figure 4:**
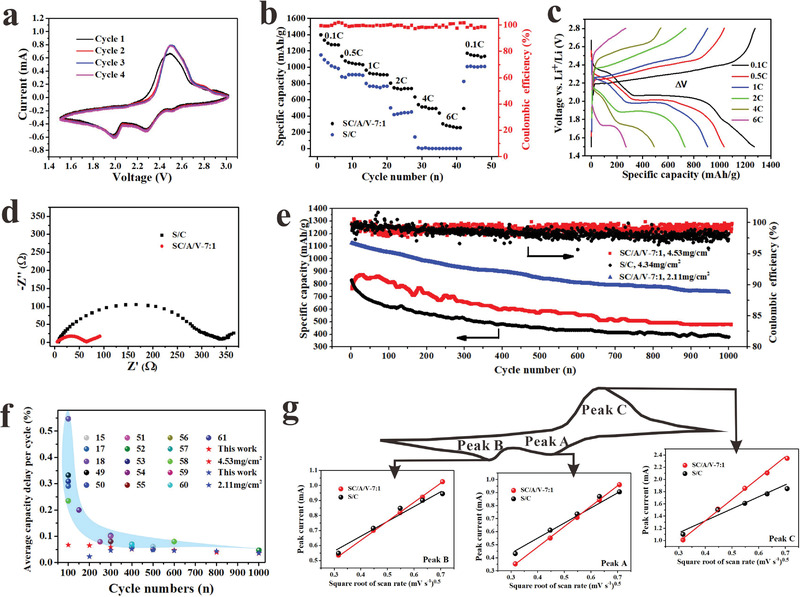
Electrochemical performance of coin cells employing the sulfur‐based cathode (SC/A/V‐7:1) with a nominal sulfur loading of ≈3 mg cm^−2^: a) CV profiles; b) C‐rate performance in comparison with that of a coin cell employing a conventional S/C cathode. c) Charge–discharge profiles at different C rates. d) Nyquist plot in comparison with that of a coin cell employing a conventional S/C composite cathode. e) Long‐term cycling stability in comparison with that of a coin cell employing a conventional S/C composite cathode at 0.5 C. f) Comparison of the capacity delay per cycle of this work with other reports with electrode skeletons. g) Linear fit of CV current for, respectively, peaks A, B, and C versus square root of scan rate.

The sulfur content in the SC/A/V‐7:1 composite electrode with a nominal sulfur loading of ≈3 mg cm^−2^ was determined to be ≈55 wt% based on thermogravimetric analysis (TGA) results (Figure S13, Supporting Information), which is a relatively high loading level for a 3D flexible cathode.^[^ ^40^, ^41^, ^42^ ^]^ For comparison, we also prepared coin cells adopting a conventional S/C composite cathode with the same nominal sulfur loading (≈3 mg cm^−2^). The cyclic voltammetry (CV) profiles of the coin cell employing the SC/A/V‐7:1 cathode after three cycles are presented in **Figure** **4**a, while the corresponding profiles obtained for the coin cell with the conventional S/C cathode are presented in the Supporting Information (Figure S14a, Supporting Information). After the first cycle, the CV profiles involving the SC/A/V‐7:1 cathode exhibit two reduction peaks at 2.31 and 2.02 V corresponding to the conversion of elemental sulfur to polysulfides and Li_2_S, respectively. Only a single intense oxidation peak is observed at 2.55 V because of the slow oxidation kinetics from Li_2_S to polysulfides, and then to elemental sulfur. We further note that the positions of peaks and the overall shape of the CV curve scarcely change after the first cycle, which verifies the good electrochemical stability of the SC/A/V‐7:1 cathode.^[^ ^43^, ^44^ ^]^ In contrast, the CV profiles involving the conventional S/C cathode exhibit poor repeatability (Figure S14a, Supporting Information). The electrochemical performances of coin cells employing the SC/A/V‐7:1 cathode and the conventional S/C cathode at different C‐rates are compared in Figure 4b. The SC/A/V‐7:1 cathode provides a high specific capacity of about 1400 mAh g^−1^ at a charge/discharge current of 0.1 C, and continuously delivers a much higher specific capacity than the conventional S/C cathode with increasing cycle numbers conducted at different C‐rates. The high specific capacity delivered by the SC/A/V‐7:1 cathode at different C‐rates can be mainly attributed to its robust, 3D porous skeleton with rapid ionic transfer capability. Moreover, after conducting increasing numbers of charge/discharge cycles with increasing C‐rates, the specific capacity delivered by the SC/A/V‐7:1 cathode recovers back to a value of 1170 mAh g^−1^ when the current returns to 0.1 C.

The above‐discussed CV profiles can be further analyzed by comparing the charge–discharge profiles featuring the voltage versus Li^+^/Li delivered by the SC/A/V‐7:1 cathode in Figure 4c as a function of its specific capacity under different C‐rates, while the corresponding results obtained for the conventional S/C cathode are presented in the Supporting Information (Figure S14b, Supporting Information). Here, it is meaningful to compare the shapes of the curve segments with reasonably stable voltages obtained at a charge/discharge current rate 0.1 C, which correspond to the reduction of elemental sulfur (S_8_) to long‐chain LiPS (2.3–2.4 V) and to the formation of short‐chain Li_2_S_2_/Li_2_S sulfide compounds (2.1 V).^[^ ^45^, ^46^ ^]^ We note that the stable voltage regions observed at 0.1 C for SC/A/V‐7:1 are more stable and exhibit a lower polarization (Δ*V*) of 0.21 V than those observed for the conventional S/C cathode (0.31 V), which suggests that the skeleton‐supported electrode provides a more kinetically efficient microenvironment. In fact, the charge–discharge profiles observed for the SC/A/V‐7:1 cathode at a high current rate of 4 C also exhibit stable voltage regions with a relatively low Δ*V*. Analysis of the Nyquist plots in Figure 4d obtained for coin cells employing the SC/A/V‐7:1 and conventional S/C cathodes based on the equivalent circuit given in the inset of the figure indicates that the charge‐transfer resistance (*R*_ct_) obtained for the SC/A/V‐7:1 cathode is much less than that obtained for the conventional S/C cathode, confirming that the conductive network constructed by the skeleton‐supported electrode provides superior electronic conductivity to that of the conventional S/C electrode.^[^ ^47^, ^48^ ^]^ Finally, the long‐term cycling stability of the Li–S coin cells employing the SC/A/V‐7:1 cathode and the conventional S/C cathode is illustrated in Figure 4e over 1000 charge/discharge cycles conducted at a current of 0.5 C. The results demonstrate that the SC/A/V‐7:1 cathode delivers excellent cycling performance. Even when the sulfur load is up to 4.53 mg cm^−2^, SC/A/V‐7:1 cathode retains a specific capacity of 485 mAh g^−1^ after 1000 cycles at 0.5 C with a Coulombic efficiency of ≈99%, which are both much greater than those delivered by the conventional S/C cathode. The above results indicate that the robust hierarchical porous skeleton greatly enhances the utilization of the AM particles over a wide range of C‐rates.

In order to further prove the excellent cycle performance of SC/A/V‐7:1 cathode, we selected recent publications about Li–S batteries that focused on electrode skeleton for further comparison (Figure 4f). The average capacity decay rate per cycle decreases with increasing cycle numbers and all the results extracted from recent publications are plotted in the shaded area.^[^ ^15^, ^17^, ^18^, ^49^, ^50^, ^51^, ^52^, ^53^, ^54^, ^55^, ^56^, ^57^, ^58^, ^59^, ^60^, ^61^ ^]^ However, it should be noted that, the capacity decay rate reported in this work turns out to be the lowest one among all of these results, which means the sample SC/A/V‐7:1 with hierarchical pores has excellent cycling stability. More details about the comparison of cycling stability are listed in Table S1 in the Supporting Information.

The excellent electrochemical performance can be attributed to the high Li^+^ diffusion coefficient and polysulfide trapping ability of SC/A/V‐7:1 electrode. To study the Li^+^ diffusion, CV measurements were performed with different scanning rates from 0.1 to 0.5 mV s^−1^ (Figure S15a,b, Supporting Information). The Li^+^ diffusion coefficient can be calculated by the classic Randles–Sevcik equation: *I*_p_ = (2.69 × 10^5^) *n* ^1.5^ *AD*_Li+_^0.5^ *Cν* ^0.5^,^[^ ^62^ ^]^ where *I*_p_ is peak current; *n* is the charge transfer number per reaction species; *A* is active electrode area; *D*_Li+_ is the Li‐ion diffusion coefficient; *C* is the concentration of lithium ions; and *ν* is the scanning rate. *n*, *A*, and *C* are the constants. Therefore, *D*_Li+_ can be determined from the slope of the fitted curve of *I*_p_ and *ν* ^0.5^. As shown in Figure 4g, the slope for SC/A/V‐7:1 electrode is larger than those for S/C electrode, implying that SC/A/V‐7:1 has a larger Li‐ion diffusion coefficient.

**Figure 5:**
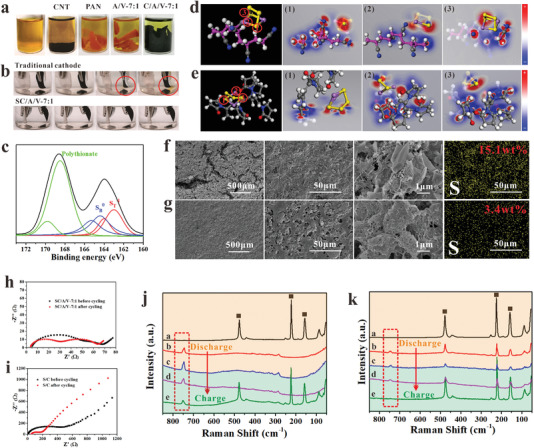
Polysulfide trapping capability of electrode samples: a) polysulfide absorption testing for MWCNT, PAN, A/V‐7:1, and C/A/V‐7:1 samples after immersion in 10 × 10^−3^ m Li_2_S_4_‐DME solutions for 3 h. b) Polysulfide trapping capability comparison for the SC/A/V‐7:1 cathode and the conventional S/C cathode under discharge at 0.5 C in a transparent liquid electrolyte. c) XPS S 2p spectra of the C/A/V‐7:1 electrode skeleton after immersion in a 10 × 10^−3^ m Li_2_S_4_‐DME solution for 3 h, and then drying. Results of MD simulations reflecting the optimized binding configurations of polysulfides with polymer chains: d) PAN chains; e) PVP chains. SEM images and EDS mapping images of cathode surfaces after 100 charge/discharge cycles at 0.5 C: f) the conventional S/C cathode; g) the SC/A/V‐7:1 cathode. Nyquist plots of a coin cell employing h) SC/A/V‐7:1 cathode and i) conventional S/C composite cathode before and after cycling (100 cycles under 0.5 C). Raman spectra for j) S/C cathode and k) SC/A/V‐7:1 cathode at representative voltage: a (pristine cathode), b (discharge to 2.28 V), c (discharge to 1.5 V), d (charge to 2.34 V), and e (charge to 3 V).

The polysulfide trapping ability of the A/V‐7:1 and C/A/V‐7:1 electrode skeletons and the SC/A/V‐7:1 composite cathode was investigated qualitatively by Li_2_S_4_ absorption testing. The possible chemical interactions between the C/A/V‐7:1 skeleton and polysulfide were analyzed quantitatively by X‐ray photoelectron spectroscopy (XPS). Finally, MD simulations were conducted to determine how the two‐component PAN/PVP binder contributes to polysulfide trapping at the molecular level. These results are presented in **Figure** **5**.

First, Li_2_S_4_ absorption testing was conducted by placing an ≈30 mg solid sample into 3 mL of a 10 × 10^−3^ m Li_2_S_4_‐DME solution, and the Li_2_S_4_ absorption ability of the sample was evaluated qualitatively according to changes in the solution color from an initial orange to a light yellow after 3 h of immersion. The initial Li_2_S_4_‐DME solution, and the solution conditions observed for MWCNT, PAN hydrogel, A/V‐7:1 hydrogel, and C/A/V‐7:1 electrode skeleton samples after 3 h of immersion are presented in Figure 5a. The results clearly demonstrate that the C/A/V‐7:1 sample exhibited the best Li_2_S_4_ absorption ability of all other samples considered. The good performance of the C/A/V‐7:1 sample over that observed for the A/V‐7:1 sample can be attributed to the microscale pores introduced on the MWCNT surfaces within the C/A/V‐7:1 skeleton. By the way, the polysulfide trapping capabilities of C/A/V‐7:1 skeleton is also the best among the C/A/V skeleton with different ratio of PAN:PVP because of the uniform micropores on MWCNTs according to the configuration snapshots of the Monte Carlo simulation of the porous structures for PAN:PVP with different ratios, as well as their polysulfide diffusion and deposition situation (Section S2 and Figures S16–S18, Supporting Information). The polysulfide trapping capabilities of the SC/A/V‐7:1 and conventional S/C cathodes can also be evaluated qualitatively by conducting a discharge cycle with the cathodes immersed in a transparent electrolyte liquid, and comparing changes in the color of the liquid during the discharge process. The results are presented over time at intervals of 1 h in Figure 5b for discharge conducted at 0.5 C. The results demonstrate that, while an orange color indicative of Li_2_S_4_ in solution is observed in the transparent electrolyte under discharge for the conventional S/C cathode, no changes are observed in the electrolyte color for the SC/A/V‐7:1 cathode.

The C/A/V‐7:1 electrode skeleton obtained after immersion in the 10 × 10^−3^ m Li_2_S_4_‐DME solution for 3 h was dried, and the sample was subjected to XPS analysis. The obtained S 2p spectrum is presented in Figure 5c, along with the results of deconvolution based on Gaussian curves, which yielded four distinct peaks. The first two located at binding energies of 164.4 and 163.0 eV can be, respectively, attributed to bridging sulfur (SB^0^) and terminal sulfur (ST^1^) in pure Li_2_S_4_.^[^ ^29^, ^63^, ^64^ ^]^ However, peaks attributable to SB^0^ and ST^1^ are also observed at slightly higher binding energies, which therefore reflect a slightly modified chemical environment for some proportion of the Li_2_S_4_ molecules. In addition, peaks are also observed at binding energies of 168.5 and 169.7 eV, which can be possibly attributed to SO*_x_* species. These results demonstrate that strong bonding interactions occur between polysulfides and the C/A/V‐7:1 skeleton, and that a proportion of the Li_2_S_4_ molecules is chemisorbed by the skeleton.

The results of MD simulations reflecting the optimized binding configurations of Li_2_S_4_ molecules with PAN chains and PVP chains are shown in Figure 5d,e, respectively, where both PAN and PVP include three different positions generating strong interactions with Li_2_S_4_. In addition, the interaction energies of Li_2_S_4_ with PAN (Δ*E*_PAN/Li2S4_) and PVP (Δ*E*_PVP/Li2S4_) were calculated as follows

(2) $$\begin{array}{c} \Delta E_{\mathrm{PAN}/\mathrm{Li}2S4}\;=\;E_{\mathrm{PAN}/\mathrm{Li}2S4}\;-E_{\mathrm{PAN}}-E_{\mathrm{Li}2S4}\\ \Delta E_{\mathrm{PVP}/\mathrm{Li}2S4}\;=\;E_{\mathrm{PVP}/\mathrm{Li}2S4}\;-{\;E}_{\mathrm{PVP}}-E_{\mathrm{Li}2S4} \end{array}$$

here *E*_P_ *_/_*_Li2S4_ is the total energy of the polymer and Li_2_S_4_ (P = PAN or PVP) system, *E*_P_ is the total energy of the polymer system, and *E*_Li2S4_ is the total energy of the Li_2_S_4_ molecule. The corresponding calculation results are also presented in Figure 5d,e. We note that both Δ*E*_PAN/Li2S4_ and Δ*E*_PVP/Li2S4_ are negative values, indicating that Li_2_S_4_ molecules spontaneously bind with both PAN and PVP chains via adsorption, as indicated by the XPS analysis, while PVP chains have a considerably stronger Li_2_S_4_ adsorption capacity than PAN chains.

The structural stability of the SC/A/V‐7:1 cathode under long‐term charge/discharge cycling can be characterized by comparing the SEM images and EDS mapping images obtained for the conventional S/C and SC/A/V‐7:1 cathode surfaces after 100 charge/discharge cycles conducted at 0.5 C, which are presented in Figure 5f,g, respectively. We note that the conventional S/C cathode surface exhibits numerous cracks and a nonuniform morphology after 100 charge/discharge cycles. Moreover, the EDS mapping results indicate that the AM particles do not remain uniformly distributed over the surface, but tend to aggregate due to LiPS deposition during cycling.^[^ ^65^ ^]^ This indicates that the AM components are not effectively fixed by the binder. In contrast, the SC/A/V‐7:1 cathode surface exhibits a uniform morphology after 100 charge/discharge cycles, and the AM particles remain uniformly distributed over the surface, which can be mainly attributed to the good polysulfide trapping capability and ability to buffer volume expansion effects of the hierarchical porous structure. This discussion is further supported by the fact that the sulfur content of the conventional S/C estimated over the imaged area is 15.1 wt% after 100 cycles, which is very much greater than the 3.4 wt% sulfur content estimated for the SC/A/V‐7:1 cathode.

To determine the resistance impedance parameters before and after cycles, the Nyquist plots of two electrodes were compared and investigated. As shown in Figure 5h,i, the value of *R*_ct_ for both electrodes exhibits an obvious decrease after 100 cycles under 0.5 C, which is mainly attributed to the activation induced by sulfur rearrangement during discharge/charge. Furthermore, compared with the Nyquist plots for cycled S/C electrode (Figure 5i), the cycled electrode for SC/A/V‐7:1 shows an extra semicircle in the mild‐frequency range in Figure 5h, which is possibly caused by the formation of the Li_2_S*_x_* layer on the cathode with stronger trapping ability of LiPS.^[^ ^66^ ^]^

In situ Raman spectra (Figure S19, Supporting Information) was used to further investigate and analyze the LiPS diffusion and deposition on S/C and SC/A/V‐7:1 cathode. As is shown in Figure 5j,k, both cathodes exhibit evident peaks at 156, 221, and 473 cm^−1^, corresponding to elemental sulfur. Following discharge, the intensity of these peaks gradually decreases. Concomitantly peaks occur due to the generation of LiPSs appear at 746 cm^−1^. During charging, these LiPSs are gradually oxidized into S_8_ and, therefore, the peaks located at 156, 221, and 473 cm^−1^ re‐appear. The peak located at 746 cm^−1^, assigned to the soluble polysulfides, is observed for both discharged cathodes. When fully charged to 2.8 V, the traditional S/C cathode continues to exhibit a peak for polysulfides, whereas the polysulfide peak for SC/A/V‐7:1 cathode (almost) disappears. Importantly, these in situ Raman results confirm that the SC/A/V‐7:1 design is significantly better than S/C cathode in regulating LiPS diffusion and deposition.

**Figure 6:**
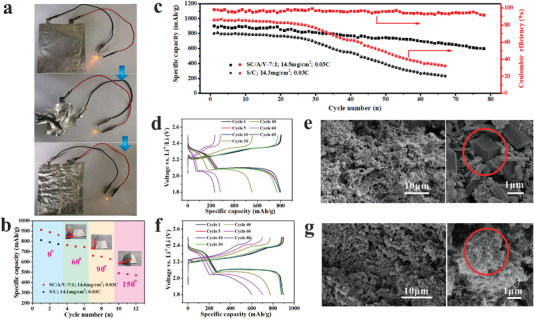
Mechanical flexibility and electrochemical performance of pouch cells employing the SC/A/V‐7:1 cathode with a high sulfur loading of 14.5 mg cm^−2^: a) demonstration of flexibility under extreme deformation conditions. b) Cycling performance in pouch cells employing conventional S/C cathodes and SC/A/V‐7:1 cathodes with different bending angles. c) Cycling performance in comparison with pouch cells employing conventional S/C cathodes. Characteristics of conventional S/C‐based pouch cells: d) charge–discharge profiles at different cycle numbers; e) cross‐sectional SEM images of the cathode after 67 cycles. Characteristics of SC/A/V‐7:1‐based pouch cells: f) charge–discharge profiles at different cycle numbers; g) cross‐sectional SEM images of the cathode after 78 cycles.

The structural flexibility of pouch cells employing SC/A/V‐7:1 cathodes with a high sulfur loading of 14.5 mg cm^−2^ can be evaluated according to the images presented in **Figure** **6**a and Movie S1 in the Supporting Information. These results demonstrate that the SC/A/V‐7:1‐based pouch cell functions properly even under harsh deformation conditions. This good flexibility can be attributed to the robust electrode skeleton that can effectively buffer applied deformations and stresses. In order to compare the electrochemical performance of SC/A/V‐7:1 and S/C cathode under external deformation, the cycle performance of SC/A/V‐7:1 cathode and traditional cathode S/C under different degrees of deformation is shown in Figure 6b. The SC/A/V‐7:1 cathode still works stably even under a rather huge deformation. In conclusion, the robust skeletons and flexibility of cathode in our work effectively stabilize the cathode structure and significantly increase the battery's resistance to large deformations from outside.

The cycling stability of the SC/A/V‐7:1‐based pouch cell with high sulfur loading is also compared with that obtained for pouch cells employing conventional S/C cathodes with a high sulfur loading of 14.3 mg cm^−2^ in Figure 6c. We note that the SC/A/V‐7:1‐based pouch cell delivers a high specific capacity around 600 mAh g^−1^ even after 78 charge/discharge cycles conducted under a current of 0.03 C, while the specific capacity of the conventional S/C‐based pouch cell decreases to less than 400 mAh g^−1^ after only 50 cycles. Moreover, the Coulombic efficiency of the SC/A/V‐7:1‐based pouch cell remains stable at greater than 95% during the entire cycling process, while that observed for the conventional S/C‐based pouch cell decreases to less than 50% after only 50 cycles.

The characteristics of conventional S/C‐based pouch cells under long‐term cycling can be further evaluated according to the charge–discharge profiles presented in Figure 6d for different cycle numbers, and the cross‐sectional SEM images presented in Figure 6e for the cathode subjected to 67 charge/discharge cycles. We note that the Δ*V* values between the stable charging and discharging voltages is very large (≈0.21 V), even in the first few cycles, which is indicative of high charge and mass transfer resistances inside the conventional cathode. In addition, the conventional S/C cathode presents regions with very high concentrations of crystalline AM components after cycling due to the high‐sulfur loading conditions and the nonuniform and poor LiPS trapping capability of the composite cathode. These results can be compared with the characteristics of SC/A/V‐7:1‐based pouch cells evaluated according to the charge–discharge profiles presented in Figure 6f for different cycle numbers, and the cross‐sectional SEM images presented in Figure 6g for the cathode subjected to 78 charge/discharge cycles. In contrast to that observed for the conventional S/C‐based pouch cell, the value of Δ*V* observed for the SC/A/V‐7:1‐based cell is only about 0.16 V, due to the fast charge and mass transport inside the hierarchical porous skeleton. In addition, the SC/A/V‐7 cathode presents no regions with high concentrations of AM components after cycling, even under the high‐sulfur loading conditions, which clearly confirms the excellent LiPS trapping capability of the hierarchical porous cathode. In addition, it is worth mentioning that the surface of lithium anode in SC/A/V‐7:1 is much smoother than that of S/C sample after cycles (Figure S20, Supporting Information). This result further reflects the improved ability of SC/A/V‐7:1 cathode to inhibit polysulfide shuttling.

## 3 Conclusion

The present work addressed the urgent need for developing cost‐effective strategies to realize robust composite electrode structures for Li–S batteries by proposing a self‐assembling electrode slurry based on a polar binder solution composed of PAN and PVP polymers with MWCNTs and graphene@sulfur AM particles that forms hierarchical porous structures via water‐vapor‐induced phase separation. The resulting 3D electrode skeleton was demonstrated both experimentally and by simulations to be a highly robust and mechanically flexible electron‐conductive network with a hierarchical porous structure composed of both macroscale and microscale pores that provides ion‐transport channels, improved electrolyte wetting, and strong polysulfide trapping capability that is further supplemented by the polar groups of the polymer binders. Composite sulfur cathodes prepared with a moderate sulfur loading of 4.53 mg cm^−2^ realized a very stable specific capacity of 485 mAh g^−1^ after 1000 charge/discharge cycles at a current of 0.5 C. Applying a composite sulfur cathode with a high sulfur loading of 14.5 mg cm^−2^ in a Li–S pouch cell with a low electrolyte:sulfur ratio of 5 mL:1 g provided good flexibility and delivered a high specific capacity of 600 mAh g^−1^ for 78 charge/discharge cycles at a current of 0.03 C. Accordingly, the results in this study demonstrate that the proposed self‐assembly strategy represents a cost‐effective and scalable strategy for realizing well‐designed and controlled porous structures in composite sulfur electrodes.

## 4 Experimental Section

### Materials

Natural graphite flakes (NG) with a purity greater than 99.9% were purchased from Shenghua Research Institute (Changsha, China). XFM04 MWCNTs were obtained with a purity >95%, lengths in the range of 0.5–2 µm, and diameters < 8 nm, and XFM13 MWCNTs with a purity >95%, lengths in the range of 10–30 µm, and diameters in the range of 10–20 nm from XFNANO Inc. (Nanjing, China). Hydrated hydrazine (purity >98.0%), DMF (purity >99.9%), sublimed sulfur powder (purity ≥99.5%), titanium butoxide (purity ≥99.0%), sulfanilic acid (purity >98%), sodium nitrite (purity 99.99%), sodium borohydride (purity 98%), sodium carbonate (purity 99.5%), and PVP powder (purity >98%, *M*_w_ = 1 300 000) were purchased from Aladdin (Shanghai, China). In addition, PAN powder (purity 99.9%, *M*_w_ = 150 000) was purchased from DOW (USA). The 1,2‐dimethoxyethane (DME) and 1,3‐dioxacyclopentane (DOL) used for the electrolyte in Li–S coin cells were purchased from Aladdin (Shanghai, China).

### Preparation of Graphene@sulfur Active Particles

Graphene@sulfur AM particles were prepared according to a previous report with little change.^[^ ^29^ ^]^ Briefly, 50 mg of sulfonated graphene (SG) and 100 mg of TiO_2_‐modified MWCNTs (ST) were added into 150 mL of distilled water, and sonication was applied for 2 h to obtain a uniform SG/ST dispersion. Then, 0.8 g of sulfur powder was dissolved in a 3.2 mL mixture of hydrazine hydrate and DMF in a 1:1 ratio by volume to obtain a homogenous brown precursor solution. Next, the full volume of the precursor solution was gradually dropped into the SG/ST dispersion (≈150 mL) along with 1 wt% H_2_O_2_ under high speed stirring (10 000 r min^−1^). Finally, the resultant dispersion was centrifuged, washed with water three times, and lyophilized to obtain the final graphene@sulfur particles.

### Preparation of 3D Porous PAN/PVP Hydrogels under Different Circumstances

Based on previous work,^[^ ^67^ ^]^ a composite PAN/PVP solution with a polymer concentration ranging from 4 to 15 wt% was prepared in DMF with PAN:PVP ratios of 3:1, 5:1, 7:1, and 9:1 by weight. Composite hydrogel samples were prepared by transferring 5 mL of the composite solution into a glass dish with a diameter of 5 cm, and then placing the dish in the enclosure with humidity *X* = 60% or 100%, a humid airflow rate of about 0.1 m^3^ s^−1^, and temperature *Y* = 0, 25, or 60 °C. The obtained PAN/PVP hydrogel samples are denoted herein as A/V‐*X*/*Y*, and samples A/V‐60/0, A/V‐60/25, A/V‐60/60, A/V‐100/0, A/V‐100/25, and A/V‐100/60 were accordingly prepared. Samples placed in a fume hood with a circulating atmosphere and *X* = 60% and *Y* = 0 or 25 °C were denoted herein as F‐A/V‐0 and F‐A/V‐25, respectively.

### Preparation of 3D Porous PAN/PVP and PAN/PVP/MWCNT Hydrogels with Different PAN:PVP Ratios

The composite hydrogels were formed by transferring 5 mL of the composite solution of DMF and PAN/PVP (5 wt%) with different PAN:PVP ratios by weight into a glass dish with a diameter (*d*) of 5 cm. Then, the dish was placed under the fume hood with a 60% relative humidity at a temperature of 20 °C for 30 min to form the PAN/PVP composite hydrogels, which was denoted herein as A/V‐1:0, A/V‐9:1, A/V‐7:1, A/V‐5:1, and A/V‐3:1 according to the PAN:PVP ratio. In addition, 100 mg MWCNT (XFM13) was added into each of the 5 mL PAN/PVP DMF composite solutions with different PAN:PVP ratios. Then, the mixture was crushed in an agate mortar for 20 min and transferred into a glass dish (*d* = 5 cm). Finally, the dish was placed under the fume hood with 60% relative humidity at a temperature of 30 °C for 30 min to form PAN/PVP/MWCNT composite hydrogels, which are denoted herein as C/A/V‐1:0, C/A/V‐9:1, C/A/V‐7:1, C/A/V‐5:1, and C/A/V‐3:1 according to the PAN:PVP ratio.

### Preparation of 3D Porous Cathodes and Conventional S/C Cathodes for Coin Cells

A mixture composed of 120 mg of SG@ST/S active particles, 20 mg of MWCNT (XFM13), and 10 mg of super‐P was crushed in an agate mortar for 30 min. Then, 1 mL of the PAN/PVP DMF composite solution (5 wt%, PAN:PVP = 7:1) was added into the agate mortar and crushing continued for another 20 min until obtaining a homogenous slurry. A thin layer of the slurry was applied onto an aluminum foil substrate, which was then placed in the fume hood with a 60% relative humidity at a temperature of 20 °C to obtain the composite SC/A/V‐7:1 hydrogel. Then, the SC/A/V‐7:1 hydrogel layer was stripped off from the aluminum foil and washed with deionized water. After lyophilization, the SC/A/V‐7:1 layer was pressed into a thin sheet under a uniform pressure of 6 MPa. Finally, the sulfur cathode disks with diameters of 12 mm and an elemental sulfur loads of about 3 mg cm^−2^ were prepared using a hole puncher.

Conventional S/C cathodes were prepared from a homogenous slurry of SG@ST/S active materials, super‐P, and poly(vinyl difluoride) with a ratio of 80:10:10 by weight in an *N*‐methyl‐2‐pyrrolidone solvent. Then, a thin layer of the slurry was uniformly applied onto an aluminum foil substrate. After drying for 12 h at 60 °C, the sulfur cathode disks with diameters of 12 mm and elemental sulfur loads of about 3 mg cm^−2^ were prepared using a hole puncher.

### Preparation of 3D Porous Cathodes and Conventional S/C Cathodes for Pouch Cells

The 3D porous cathodes were prepared by applying a layer of the homogenous self‐assembling slurry onto a glass plate, and then thin aluminum mesh (7 cm × 7 cm) was placed flat on the slurry gently. Another layer of slurry was applied to cover the aluminum mesh. The electrode skeleton was formed by placing the electrode assembly under the fume hood at 60% relative humidity at a temperature of 20 °C for 30 min. The obtained electrode assembly was immersed in deionized water for 24 h to remove the DMF. Finally, the skeleton was lyophilized and subjected to a uniform pressure of 6.0 MPa for 5 min to obtain electrode materials of uniform thickness. These materials were used to assemble pouch cells with a high sulfur loading (≈14.5 mg cm^−2^) and low electrolyte/sulfur ratio (5 mL:1 g).

### Characterization

The morphology of the composite hydrogel samples was characterized by field‐emission SEM (FESEM) using a JOEL JSM‐5900LV microscope (FESEM, Japan) at an acceleration voltage of 20 kV, and EDS mapping was conducted using an Ultim Extreme detector (Oxford Instruments). The mechanical response of the composite hydrogel samples was evaluated at a temperature of 25 °C by dynamic rheological testing conducted using an AR2000EX rotational rheometer (TA instruments, USA) with parallel‐plate geometry and a diameter of 25 mm. The tensile properties of C/A/V electrodes with different PAN:PVP ratios were tested using an Instron 5967 universal tester (USA) at a cross‐head speed of 2 mm min^−1^. Five rectangular specimens (length × width × thickness: 50 mm × 5 mm × 6 mm) of each kind of electrodes were tested and the averaged values were reported. Dynamic thermomechanical analyzer (TAQ 800 instrument) was used to investigate the robustness of C/A/V electrodes with different PAN:PVP ratios. Dynamic mechanical analysis (DMA) was performed at a frequency of 1 Hz and a heating rate of 3 °C min^−1^ in a temperature range from room temperature to 45 °C. The hardness of C/A/V electrodes with different PAN:PVP ratios was tested by the Shore hardness tester (LX‐A, China). The SSA and pore size distribution of the composite hydrogel samples were obtained by BET measurements in conjunction with N_2_ adsorption/desorption analyses conducted at a temperature of 77 K using an Autosorb iQ/ASiQ adsorption analyzer (Quantachrome, USA). XPS characterization was conducted using an XSAM800 spectrometer (Kratos Company, UK) with AlK*α* radiation (*hv* = 1486.6 eV). The sulfur content in the final composites was measured by TGA conducted using a Q600 analyzer (TA instrument, USA) at a heating rate of 10 °C min^−1^ under a N_2_ atmosphere. The volume electrical resistance of composite samples having dimensions of 20 mm × 10 mm × 0.1 mm was measured using a 6517B type resistance meter when the sample resistance was less than 10^9^ Ω cm, while the volume resistance was measured using a ZC‐36 high resistance meter for sample resistances greater than 10^9^ Ω cm. Conductive silver glue was applied on both ends of the sample to reduce the contact resistance between the electrodes and the sample.

The polysulfide trapping capability of electrode skeleton samples was evaluated experimentally as follows. First, a Li_2_S_4_ solution (10 × 10^−3^ m) was prepared by reacting Li_2_S and elemental sulfur with a molar ratio of 1:3 in an anhydrous DME solvent at a temperature of 60 °C. Then, 30 mg of XFM13 MWCNTs, A/V‐7:1, and C/A/V‐7:1 samples were separately added to glass vials containing 3 mL of the Li_2_S_4_ solution (10 × 10^−3^ m), and allowed to remain immersed for 3 h. The Li_2_S_4_ absorption ability of the samples was evaluated qualitatively according to changes in the solution color from an initial orange to a light yellow over time. All procedures were completed in an Ar‐filled glovebox.

### Electrochemical Performance and Evaluation of Polysulfide Trapping

The electrochemical performance of the electrodes was evaluated using 2032‐type coin cells. The electrolyte contained 1 m LiTFSI and 1 wt% LiNO_3_ in a ratio of 1:1 by volume, DME, and DOL. The diameter and thickness of the lithium anode used in the coin cells were 12 mm and 0.5 mm, respectively. The size and thickness of the lithium anode used in the pouch cells were 60 × 75 mm and 0.5 mm, respectively. The charge/discharge behaviors of the cells at different current densities as a function of their maximum capacities (i.e., C rates) and their long‐term cycling characteristics were evaluated at a temperature of 25 °C using a NEWARE battery tester (Neware Electronics Co., Ltd., China) over a voltage range between 1.5 and 2.8 V. The specific capacity was calculated based on the mass of elemental sulfur. CV was conducted on a CHI600E electrochemical workstation (ChenHua Instruments Co., Ltd., China) at a scan rate of 0.2 mV s^−1^ between 1.5 and 2.8 V, and the Nyquist plots of the electrodes were recorded in the frequency range of 10 mHz to 100 kHz. The cycling performance obtained with high sulfur loading was evaluated using pouch cells with a low electrolyte:sulfur ratio of 5 mL to 1 g. Cell activation was initially conducted under a charge/discharge current of 0.01 C, and cycle testing was conducted under a rate of 0.03 C until being stopped automatically.

### Simulations

Interactions between PAN and PVP were evaluated based on the results of MD simulations conducted with PAN chains, PVP chains, and mixed PAN and PVP chains composed of five structural units each using the CASTEP module of Materials Studio 2019 (Dassault Systèmes).^[^ ^32^ ^]^ In addition, MD simulations were applied to determine the polysulfide adsorption capability of the composite hydrogels. The polysulfide adsorption energy (Δ*E*) was calculated according to Δ*E* = *E*_Total_ − (*E*_PAN/PVP_ + *E*_PS_), where *E*_Total_ is the total energy of the system with polysulfide adsorption, and *E*_PAN/PVP_ and *E*_PS_ are the separate formation enthalpies of the PAN, PVP, and the polysulfide, respectively. Electron cloud distribution images were also obtained through the corresponding CASTEP module.

The effects of the PAN–PVP interaction energies obtained from MD simulations on the formation of co‐continuous microphase structures in PAN/PVP/DMF systems were also evaluated by a previously proposed lattice‐based Monte Carlo simulation method^[^ ^33^, ^34^ ^]^ combined with the bond fluctuation model. This combined model was extensively applied to bulk polymer melts^[^ ^33^, ^34^, ^35^ ^]^ and polymer blends^[^ ^36^, ^37^, ^38^, ^39^ ^]^ due to its computational efficiency. The simulations employed a constant volume cubic box with dimensions of 120 × 120 × 120 under periodic boundary conditions (here all lengths are expressed in units of lattice spacing), within which the molecular motions of 109 polymer A (PAN) chains composed of 180 monomers, 959 polymer B (PVP) chains composed of 300 monomers, and 556 680 molecules C (DMF) were simulated at *k*_B_ *T* = 1, where *k*_B_ is the Boltzmann constant and *T* is the temperature. This represented an A:B ratio of 7:1 by weight according to the average densities of the polymers. The lattice‐based Monte Carlo simulations were conducted by choosing a monomer or DMF molecule at random and attempting to move it to one of its six nearest neighbors. An attempted move was accepted if it obeyed the excluded volume constraint, where only a single bead could occupy a single lattice site, and the polymers obeyed the chain connectivity constraint, which restricted the lengths of bonds connecting two neighboring monomers to the set of values {1, $\sqrt{2}$, $\sqrt{3}$}. Subsequently, the effect of MWCNTs on the phase‐separation behavior of the PAN/PVP binder system was evaluated by simulating the AB system with the bottom plane of the system in contact with a thin MWCNT layer, and the interaction energy between polymers A and B and the MWCNT layer were defined according to the distance between the polymer chains and the MWCNT layer, which varied from −2 to 0 with increasing distance.

## Conflict of Interest

The authors declare no conflict of interest.

## Supporting information

Supporting Information

Supplemental Movie 1

## Data Availability

Research data are not shared.
