# Supplementary material for: High‐Performance Flexible Sulfur Cathodes with Robust Electrode Skeletons Built by a Hierarchical Self‐Assembling Slurry
Source: Adv Sci (Weinh). 2022 Jul 19;9(26):2201881. doi: 10.1002/advs.202201881 (PMC9475518; doi:10.1002/advs.202201881)
Supplement: Supplementary file 1 — Supporting Information [file ADVS-9-2201881-s001.pdf]

ADVANCED SCIENCE
Open Access

Supporting Information

for *Adv. Sci.*, DOI 10.1002/advs.202201881

High-Performance Flexible Sulfur Cathodes with Robust Electrode Skeletons Built by a Hierarchical Self-Assembling Slurry

Zhengmin Zhang, Jiangyang Mo, Peng Yu, Lanxiang Feng, Yu Wang, Yuyuan Lu and Wei Yang**

Supporting Information

High-Performance Flexible Sulfur Cathodes with Robust Electrode Skeletons Built by a Hierarchical Self-Assembling Slurry

Zhengmin Zhang, Jiangyang Mo, Peng Yu, Lanxiang Feng, Yu Wang, Yuyuan Lu*, Wei Yang*

SI-1

The scaffold structure can form only when the polymer solution concentration is greater than 4 wt%. Therefore, the influence of the other factors, including temperature and relative humidity, was evaluated by forming composite scaffolds with a 5 wt% PAN/PVP DMF solution under different conditions. The structures of the obtained scaffolds are indicated by the representative SEM images presented in Figures S2a–h. The results indicate that high temperatures greatly increase the speed of self-assembly, and the overly fast kinetic process impedes the formation of complete porous structures (Figures S2e and h). As a result, the mechanical strength of the A/V-60/60 and A/V-100/60 samples is poor (Figure S3), particularly, that of sample A/V-100/60. At temperatures less than 60°C, the pore size increases with increasing relative humidity. This is because high humidity induces the precipitation of PAN/PVP on the surface of the PAN/PVP DMF solution, and the PAN/PVP precipitates will greatly increase the self-assembly speed of PAN/PVP inside the solution. This discontinuous self-assembly process occurring under high relative humidity also produces scaffolds with a relatively low mechanical strength (Figure S3). Accordingly, 3D porous scaffolds with high mechanical strength can be formed only under relatively low temperature with the appropriate humidity (Figures S2a–d). However, the time required to form the scaffold becomes excessively long when the temperature is too low (Figures S2a and c). Therefore, a temperature of 25°C and a relative humidity of 60% are selected as the optimal conditions. The results further

demonstrate that the application of a circulating atmosphere during sample formation will effectively decrease the time required for building the scaffold, and decrease the pore size as well (Figures S2a and b). This also increases the mechanical strength of the samples (Figure S3) because the circulating atmosphere increases the rate of DMF solvent evaporation, which in turn increases the flow state of the composite solution. The more intense flow in the solution will induce a greater number of tangles between the polymer chains, which is responsible for the decreased pore size observed under the circulating atmosphere compared with an equivalent relative humidity without the circulation (Figures S2a–d). We further note that the storage modulus of the F-A/V-25 composite scaffold maintains constant values with increasing frequency (Figure S3), which clearly indicates that stable network structures were formed in the scaffold.

In addition to the above-discussed environmental factors, the molar PAN: PVP ratio in the DMF solution was found to be another critical factor greatly influencing the pore structures of the resulting electrode skeletons. Without PVP, the PAN-DMF binder ultimately generated an open sponge-like pore network structure under the optimal environmental conditions (Figure S4a). However, the pore structure progressively transformed into a honeycomb-like configuration with increasing PVP content (Figures S4b–e), and the pore size increased as well (Figures S4c–e). Therefore, the average size of the honeycomb-like macroscale pores is the smallest for a PAN: PVP ratio of 7:1. This sample has the best mechanical performance as well (Figure S5). Accordingly, a PAN: PVP ratio of 7:1 was applied as the optimal ratio.

The effects of MWCNT content on the pore structures of the prepared electrode skeletons were also investigated. The results demonstrate that the porous structures of the A/V-7:1 sample were not notably affected by the addition of MWCNTs (Figures S6a–c). Mechanical properties of samples with different ratios of PAN and PVP were also investigated. Firstly, Figure S7a displays the representative tensile stress-strain curves, and the tensile strength and elongation at break are summarized in Figure S7b. As shown in Figure S7b, C/A/V-7:1 electrode possesses the highest tensile strength of ca. 0.44 ± 0.02 MPa and the largest strain-at-failure of ca. 28.9 ± 7.6 %.

Secondly, dynamic thermomechanical analyzer (TAQ 800 instrument) was used

to investigate the robustness of C/A/V electrodes with different PAN:PVP ratios. As shown in Figure S7c, after the introduction of PVP, the storage modulus of electrodes improves evidently due to the exacerbated polymer network entanglements. However, benefiting from a complete network structure, C/A/V-7:1 electrode shows a high storage modulus and possesses better robustness with temperature.

Thirdly, rheological measurements of C/A/V electrodes with different PAN:PVP ratios were tested by a stress-controlled rotational rheometer with a dynamic frequency sweep from 0.01 to 100 Hz at a strain of 0.5%. As shown in Figure S7d, the storage modulus of all electrodes improves with the increase of frequency. The C/A/V-7:1 electrode exhibits a larger storage modulus than those of C/A/V-1:0, C/A/V-9:1, and C/A/V-5:1 electrodes.

In addition, the hardness of C/A/V electrodes with different PAN:PVP ratios was tested by the Shore hardness tester. As shown in Figure S7e, due to the introduction of PVP, the hardness of electrodes improves slightly.

In summary, the C/A/V-7:1 electrode presents robust mechanical properties and excellent flexibility.

Moreover, MWCNTs tend to concentrate at the surface of the PAN/PVP skeleton, and thereby provide flexible electronic conductive pathways. The electronic conductivity of the nanocomposite skeleton was further optimized (Figure S8a), and the C/A/V-7:1 sample provided the best conductivity because of its small pore size and dense conductive network. Also, the C/A/V-7:1 had the highest SSA (Figure S8b) because of the homogeneous micropore on MWCNTs.

SI-2

We provided configuration snapshots of the Monte Carlo simulation of the porous structures for PAN:PVP with different ratios, as well as their polysulfide diffusion and deposition situation. As shown in Figure S16, the size of the micro-pores becomes larger with the increasing proportion of PVP. To show the LiPS trapping capability more intuitively, as shown in Figure S17, we present the configuration snapshots of 500000 LiPS particles in different C/A/V composites at

time t =0 and t = 5000. The number of LiPS particles that diffuse out of the C/A/V composites ranks as C-A-1:0 > C-A-3:1 > C-A-5:1> C-A-9:1 > C-A-7:1 (Figure S18), which confirms that the LiPS trapping capability of C/A/V-7:1 composite is better than other proportions. The LiPS trapping capability of C/A/V-7:1 composite is better than that of C/A/V-3:1 composite and C/A/V-5:1 composite because the size of the micro-pores is smaller. However, when comparing C/A/V-7:1 with C/A/V-9:1, the loading of PVP in the composite becomes a dominating factor as PVP can chemically trap more LiPS than PAN (see Figures 5 d and e), which explains that the LiPS trapping capability of C/A/V-9:1 composite is worse than that of C/A/V-7:1 composite.

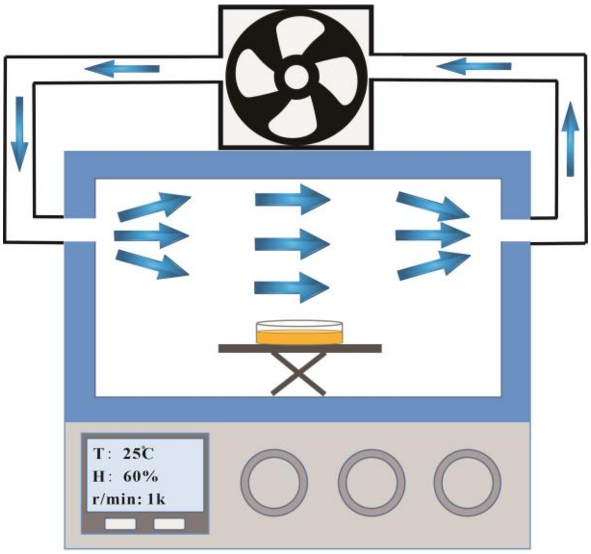

Figure S1. Schematic illustrating the fume hood device employed for preparing composite cathode electrodes via the controlled input of water vapor.

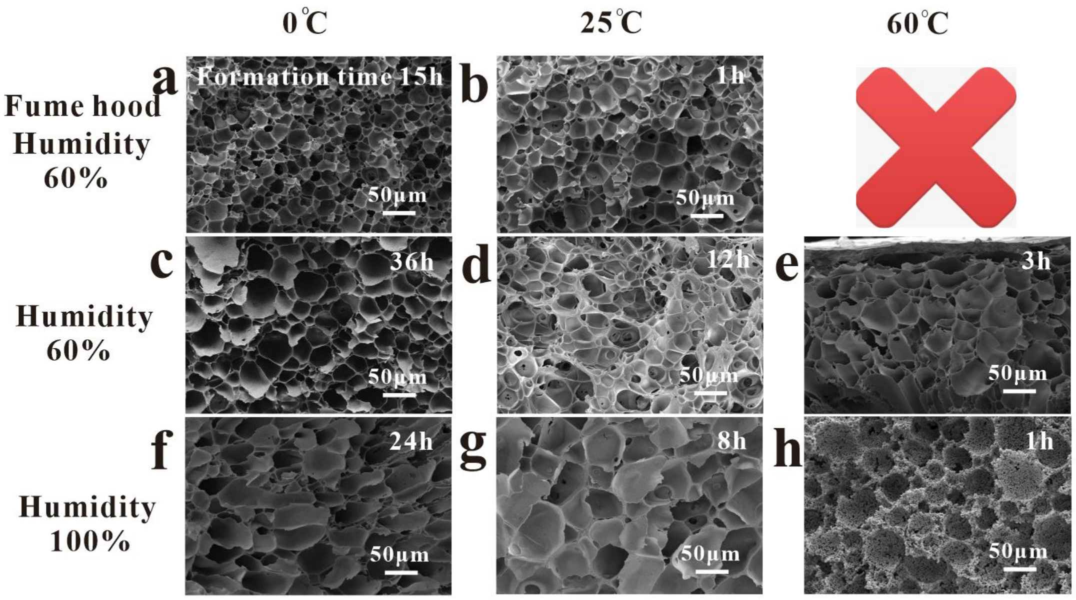

Figure S2. SEM images of (a) F-A/V-0, (b) F-A/V-25, (c) A/V-60/0, (d) A/V-60/25, (e) A/V-60/60, (f) A/V-100/0, (g) A/V-100/25, and (h) A/V-100/60 samples. The time required to form the observed scaffold is denoted in each image. In addition, a circulating atmosphere was applied in the fume hood, but not otherwise.

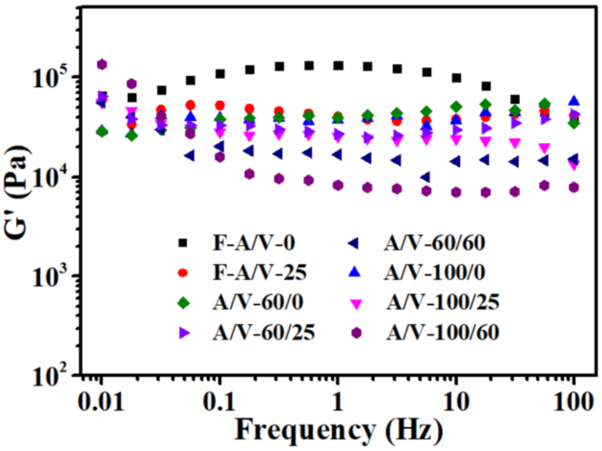

Figure S3. Storage modulus values of F-A/V-0, F-A/V-25, A/V-60/0, A/V-60/25, A/V-60/60, A/V-100/0, A/V-100/25 and A/V-100/60 samples.

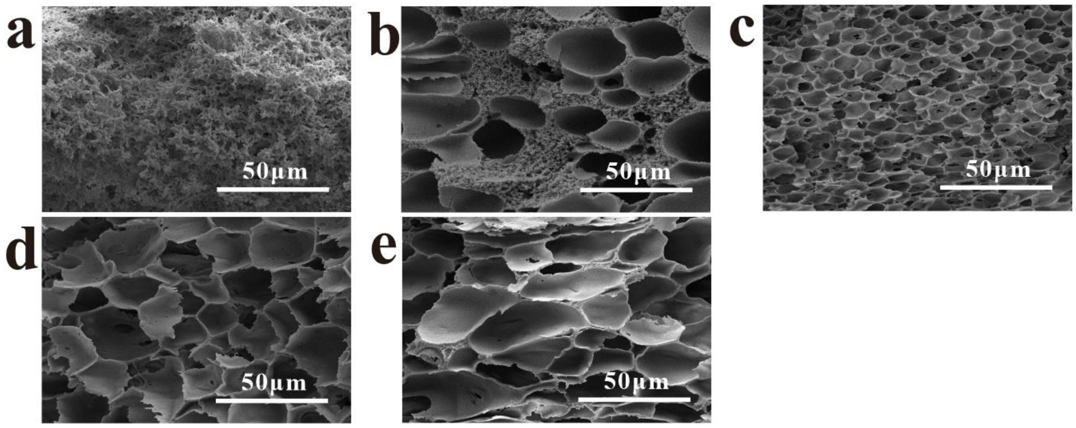

Figure S4. SEM images of (a) A/V-1:0, (b) A/V-9:1, (c) A/V-7:1, (d) A/V-5:1, (e) A/V-3:1 samples.

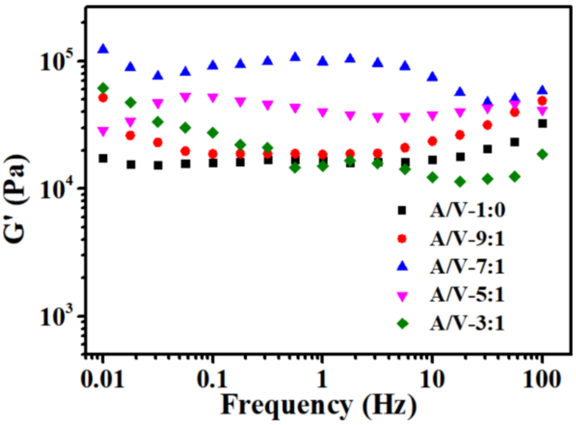

Figure S5. Storage modulus values of (a) A/V-1:0, (b) A/V-9:1, (c) A/V-7:1, (d) A/V-5:1, and (e) A/V-3:1 composite scaffold.

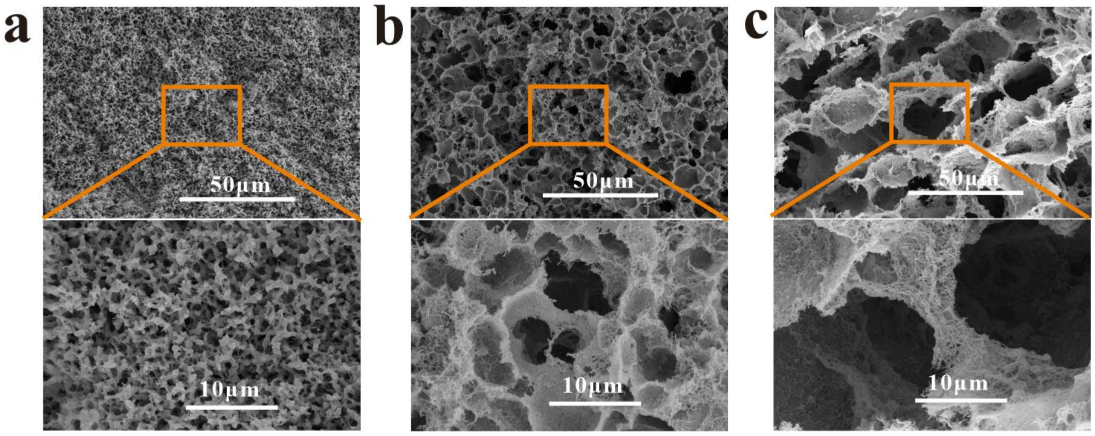

Figure S6. SEM images of (a) C/A/V-1:0, (b) C/A/V-7:1, and (c) C/A/V-5:1 samples.

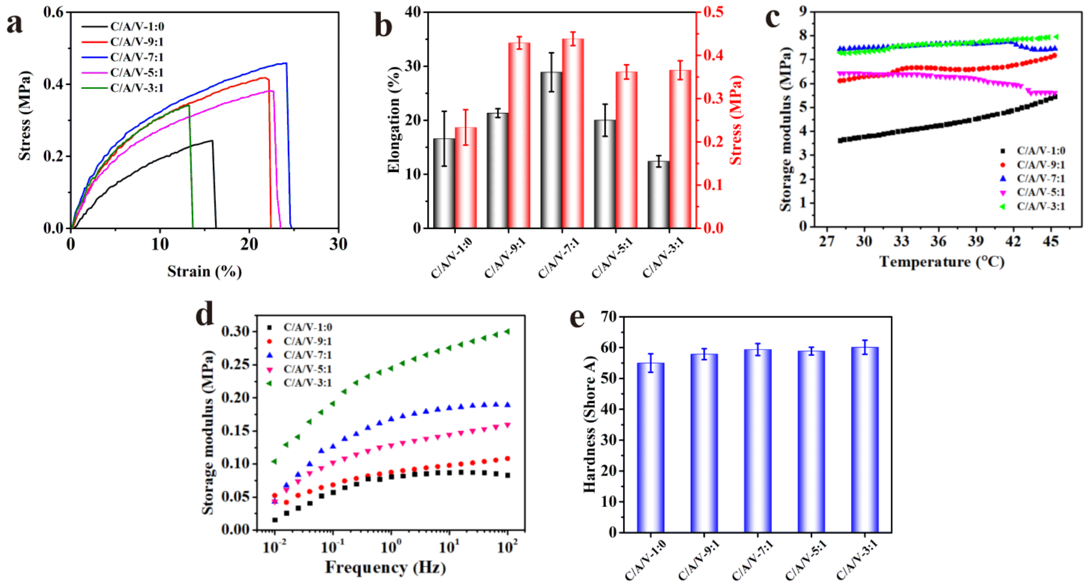

Figure S7. Mechanical properties of C/A/V electrodes (a) tensile stress-strain curves, (b) summarized tensile strengths and elongations at break, (c) storage modulus curves with the increase of temperature, (d) dynamic frequency sweep curves, and (e) hardness of C/A/V electrodes.

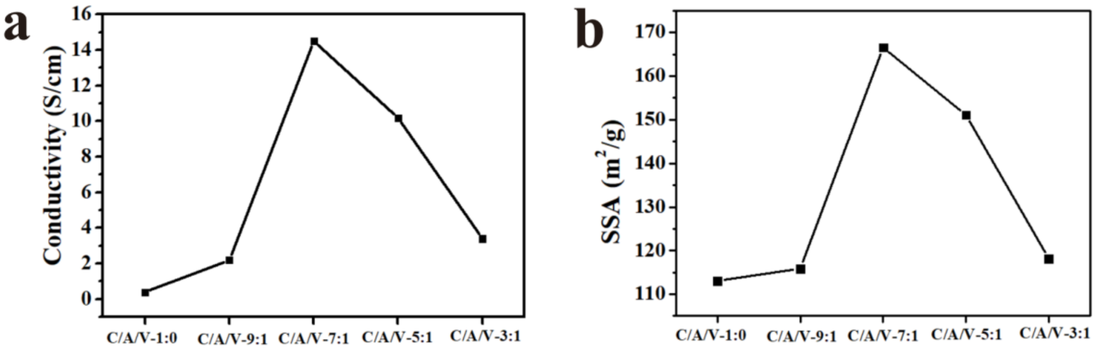

Figure S8. (a) Conductivity of C/A/V-1:0, C/A/V-9:1, C/A/V-7:1, C/A/V-5:1 and C/A/V-3:1 cathode electrodes. (b) SSA of sample C/A/V composites with different ratio of PAN:PVP.

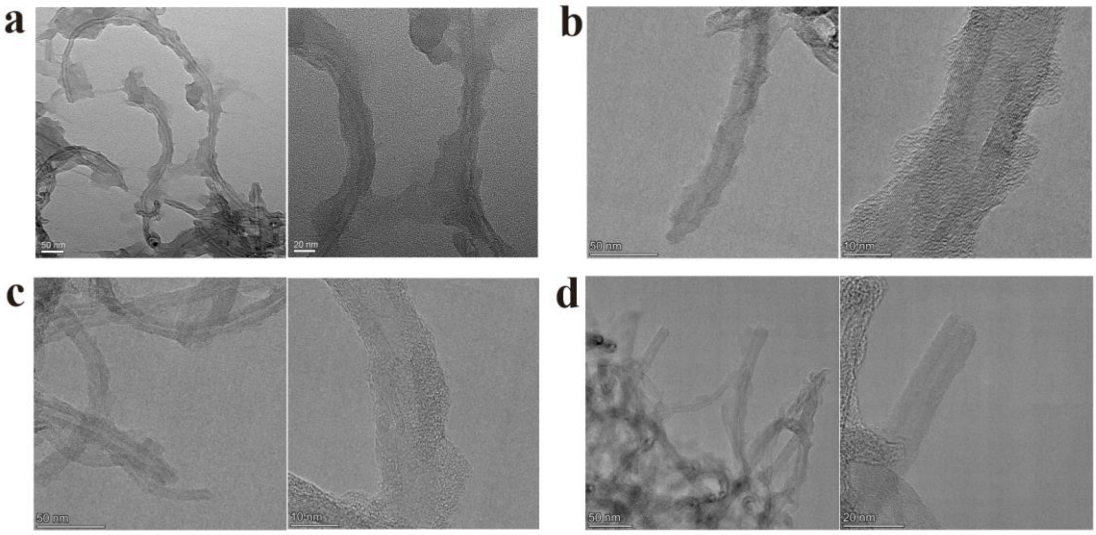

Figure S9. TEM images of (a) C/A/V-1:0, (b) C/A/V-9:1, (c) C/A/V-5:1, (d) C/A/V-3:1.

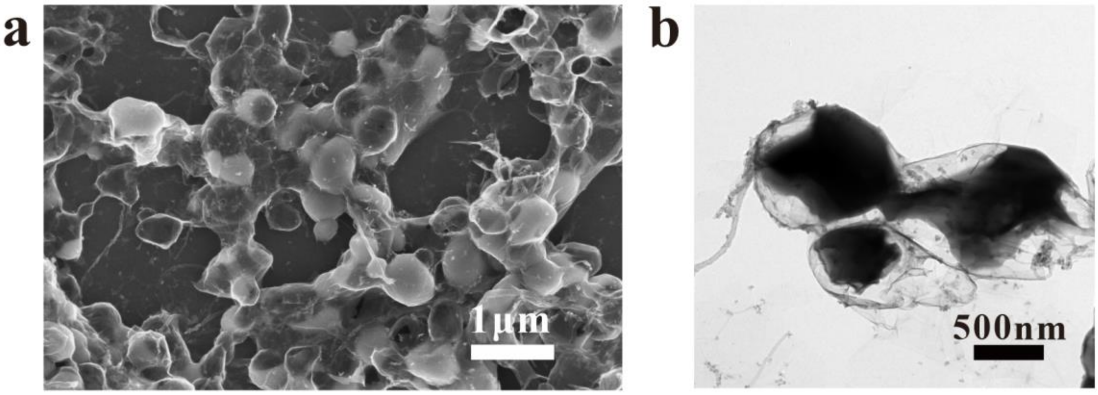

Figure S10. SEM and TEM images of active material.

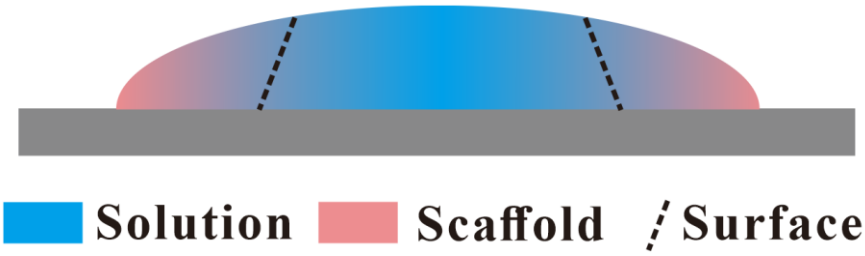

Figure S11. Schematic illustrating the formation process of the A/V-7:1 structure from a drop of the corresponding composite solution.

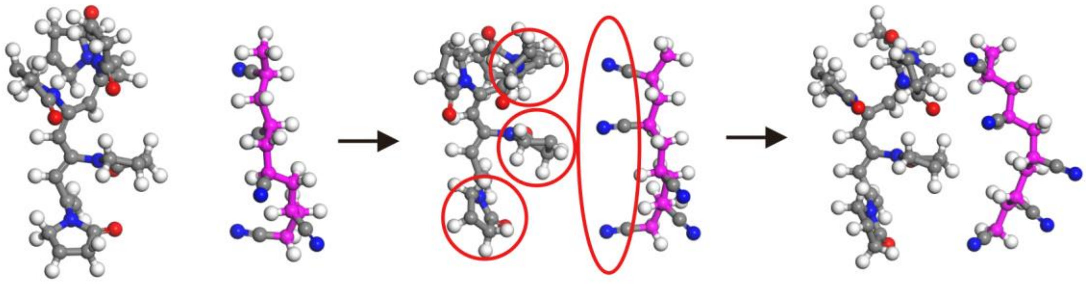

Figure S12. MD simulations of the kinetic optimization process of PAN/PVP system.

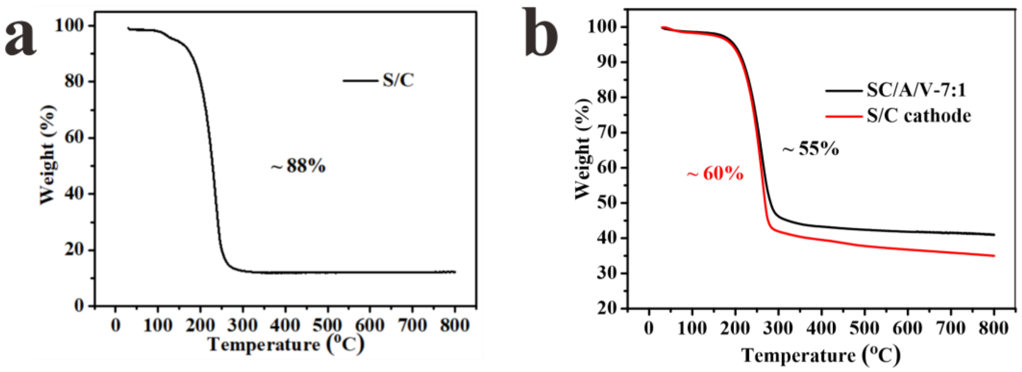

Figure S13. TG curves of (a) S/C, and (b) SC/A/V-7:1 and S/C cathode samples.

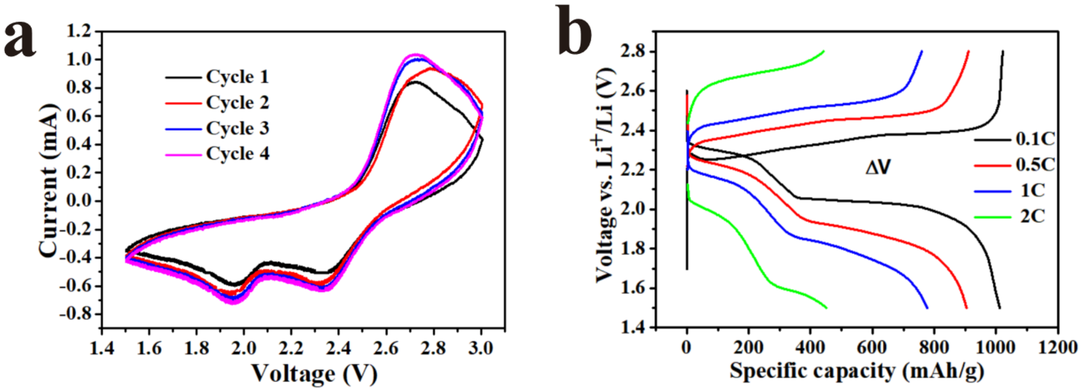

Figure S14. (a) CV profiles of the S/C cathode. (b) Charge-discharge profiles of the S/C cathode under different C rates.

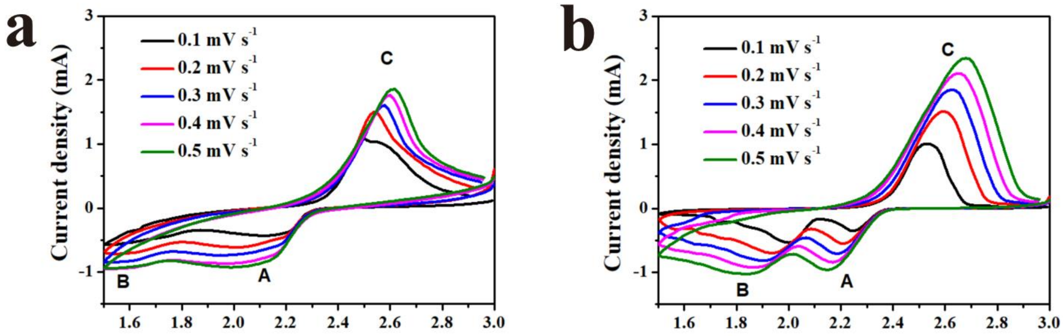

Figure S15. CV profiles for (a) S/C and (b) SC/A/V-7:1 electrode at differing scan rate.

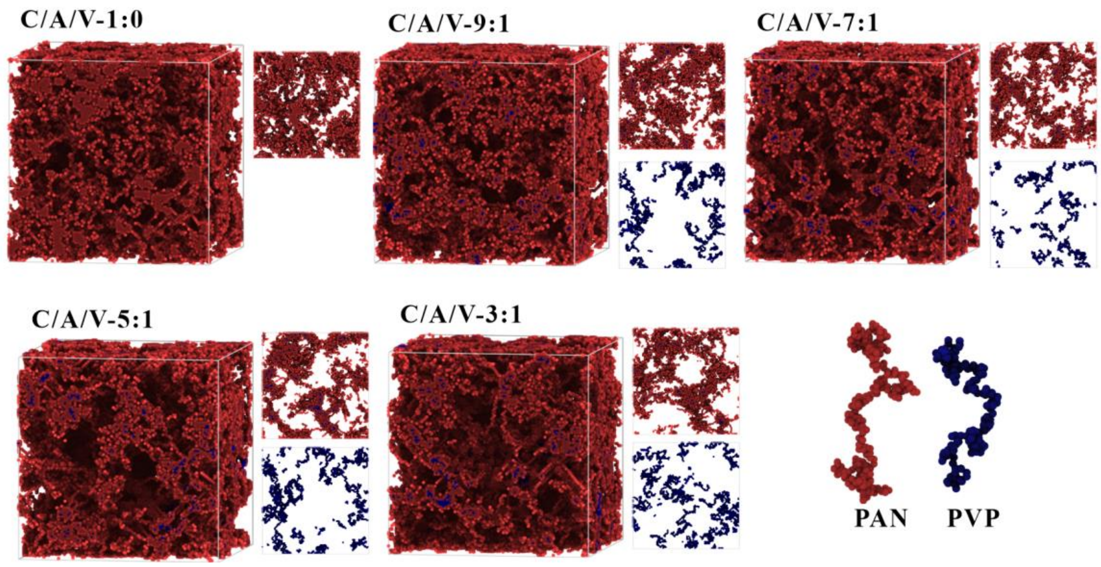

Figure S16. Configuration snapshots of Monte Carlo simulations for different structures of sample C/A/V with different ratios of PAN:PVP.

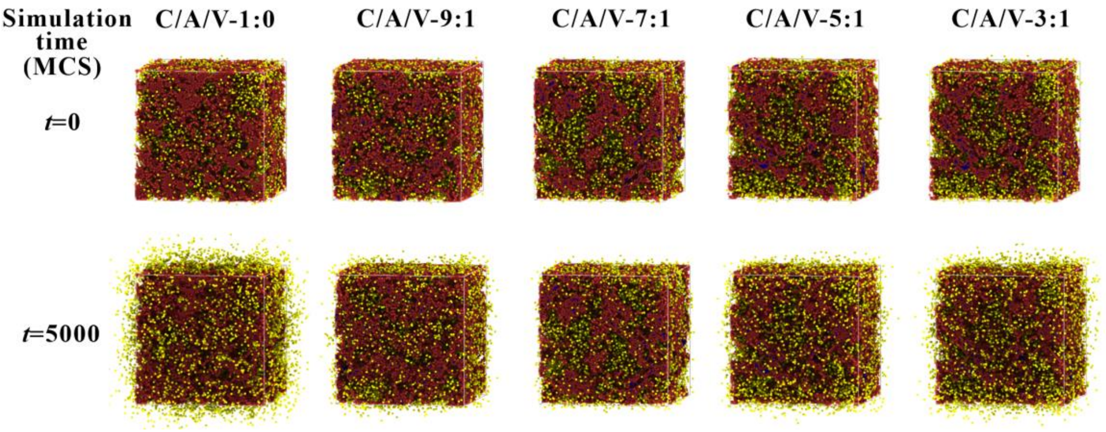

Figure R17. Configuration snapshots of 500000 LiPS particles in different structures of sample C/A/V with different ratios of PAN:PVP.

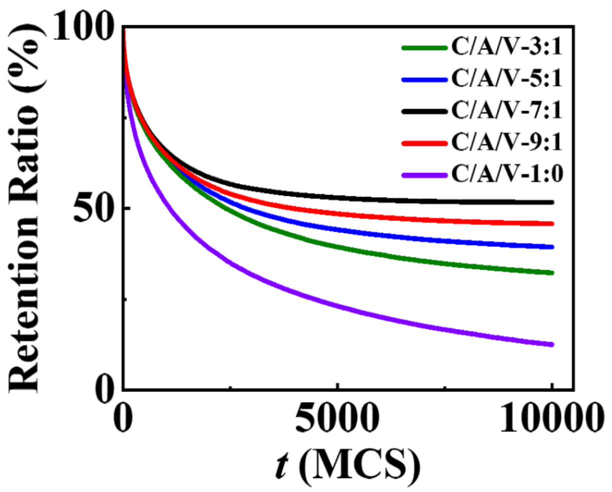

Figure S18. Retention ration of LiPS of Monte Carlo simulations in different structures of sample C/A/V with different ratios of PAN:PVP.

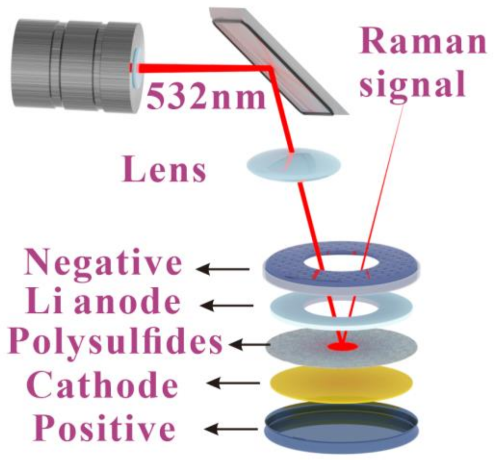

Figure S19. Schematic of cell configuration used for in-situ Raman measurement.

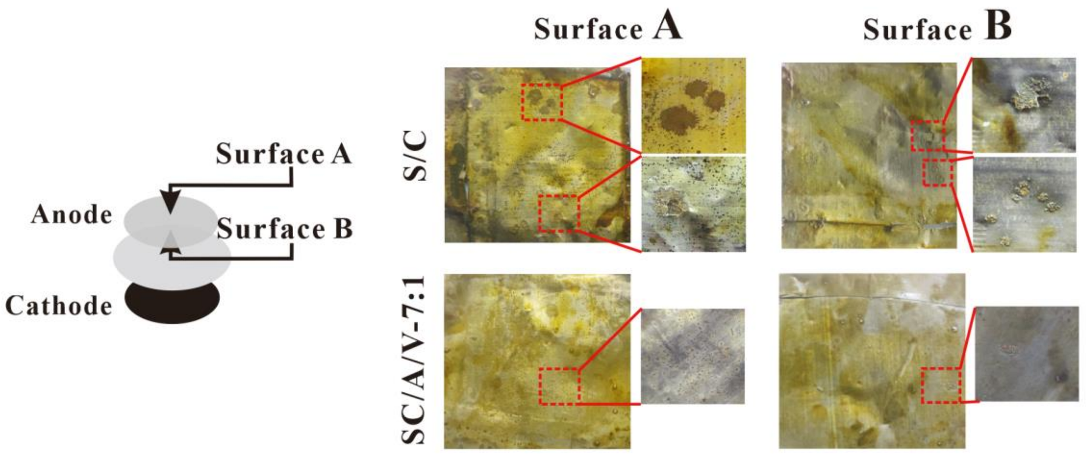

Figure S20. Digital pictures of both sides of Li anode in a pouch cell employing (a) SC/A/V-7:1 cathode and (b) conventional S/C composite cathode after cycling.

Table S1. A comparison of the cycling stability between this work and other publications of the electrode skeletons

Electrode skeleton types	C-rate	cycle number	S loading (mg/cm^2)	Capacity Retention (mAh/g)	Capacity fading rate per cycle(%)	Reference
S-Nb_2O_5−x/CNTs	1	500	2	847	0.050	17
P(VDF-TRFE)	0.2	100	N/A	801	0.333	49
3D N-doped graphene foam	2	500	2.05	440.3	0.061	50
PVDF-HFP	0.5	300	2	498.9	0.103	51
Single-wall carbon nanotube network	0.15	300	2.4	908	0.100	52
3D interconnected carbon nanotube foam	0.2	1000	0.9	713	0.047	53
S-PCNT	0.1	100	1.8	526	0.309	54
3D Carbon Nanotube Foam	0.1	100	19.1	471	0.547	55
Carbon-Nanotube Paper	0.05	150	6.3	696.5	0.200	56
CNF/S/PANi	0.2	300	1	953	0.080	57
PPA	1.5	400	1.5	430	0.070	58
cellulose-derived carbon	0.5	400	1.3	1000	0.058	59
RuO_2-x	0.2; 0.2	100; 600	5.5; 5.5	978; 602	0.235; 0.080	60
PCF/VN/S	0.1	250	8.1	1052	0.079	61
S-CNTs/NCNFs/PVDF	0.2	400	2	933	0.069	62
Phosphorus-Doped carbon	0.1	100	8.2	850	0.291	63
PAN/PVP/CNT	0.5	1000	4.53; 2.11	485; 739	0.036; 0.035	This work
